# Sex-Specific Effects of Prenatal Hypoxia and a Placental Antioxidant Treatment on Cardiac Mitochondrial Function in the Young Adult Offspring

**DOI:** 10.3390/ijms241713624

**Published:** 2023-09-03

**Authors:** Paulami Chatterjee, Claudia D. Holody, Raven Kirschenman, Murilo E. Graton, Floor Spaans, Tom J. Phillips, C. Patrick Case, Stephane L. Bourque, Hélène Lemieux, Sandra T. Davidge

**Affiliations:** 1 Department of Physiology, University of Alberta, Edmonton, AB T6G 2R3, Canada; paulami@ualberta.ca; 2 Department of Obstetrics and Gynecology, University of Alberta, Edmonton, AB T6G 2R3, Canada; raven@ualberta.ca (R.K.); graton@ualberta.ca (M.E.G.); floortje@ualberta.ca (F.S.); 3 Women and Children’s Health Research Institute, University of Alberta, Edmonton, AB T6G 2R3, Canada; sbourque@ualberta.ca (S.L.B.); hlne@ualberta.ca (H.L.); 4 Faculty Saint-Jean, University of Alberta, Edmonton, AB T6G 2R3, Canada; holody@ualberta.ca; 5 Department of Pediatrics, University of Alberta, Edmonton, AB T6G 2R3, Canada; 6 UK Dementia Research Institute, Cardiff University, Cardiff CF10 3AT, UK; tp12155@my.bristol.ac.uk; 7 Musculoskeletal Research Unit, University of Bristol, Bristol BS10 5NB, UK; pacpc@my.bristol.ac.uk; 8 Department of Anesthesiology & Pain Medicine, University of Alberta, Edmonton, AB T6G 2R3, Canada; 9 Department of Medicine, University of Alberta, Edmonton, AB T6G 2R3, Canada

**Keywords:** mitochondria, oxidative phosphorylation (OXPHOS), cardiac, prenatal hypoxia, nMitoQ treatment, offspring, developmental origins of health and disease (DOHaD)

## Abstract

Prenatal hypoxia is associated with placental oxidative stress, leading to impaired fetal growth and an increased risk of cardiovascular disease in the adult offspring; however, the mechanisms are unknown. Alterations in mitochondrial function may result in impaired cardiac function in offspring. In this study, we hypothesized that cardiac mitochondrial function is impaired in adult offspring exposed to intrauterine hypoxia, which can be prevented by placental treatment with a nanoparticle-encapsulated mitochondrial antioxidant (nMitoQ). Cardiac mitochondrial respiration was assessed in 4-month-old rat offspring exposed to prenatal hypoxia (11% O_2_) from gestational day (GD)15–21 receiving either saline or nMitoQ on GD 15. Prenatal hypoxia did not alter cardiac mitochondrial oxidative phosphorylation capacity in the male offspring. In females, the NADH + succinate pathway capacity decreased by prenatal hypoxia and tended to be increased by nMitoQ. Prenatal hypoxia also decreased the succinate pathway capacity in females. nMitoQ treatment increased respiratory coupling efficiency in prenatal hypoxia-exposed female offspring. In conclusion, prenatal hypoxia impaired cardiac mitochondrial function in adult female offspring only, which was improved with prenatal nMitoQ treatment. Therefore, treatment strategies targeting placental oxidative stress in prenatal hypoxia may reduce the risk of cardiovascular disease in adult offspring by improving cardiac mitochondrial function in a sex-specific manner.

## 1. Introduction

Cardiovascular diseases, accounting for over 30% of all fatalities worldwide (2019), are recognized as a leading cause of death globally by the World Health Organization [1]. Behavioral risk factors associated with cardiovascular disease, such as sedentary behavior, poor nutrition, or smoking, can be controlled through lifestyle changes [2,3,4]. However, research for the past few decades has shown that cardiovascular disease risk throughout life is also influenced by developmental programming [5], which could be prevented by early intervention strategies. Indeed, offspring exposed to an unfavorable intrauterine environment in complicated pregnancies are found to be at increased risk of cardiovascular morbidities in adulthood compared to offspring from normal pregnancies (reviewed in [6]). Pregnancy complications such as preeclampsia, placental insufficiency, or umbilical cord abnormalities are often associated with a low oxygen environment in utero [7,8,9] (leading to fetal hypoxia), thereby impairing normal fetal growth and development. Prenatal exposure to hypoxia has been shown to compromise cardiac development in the fetus and lead to long-term adverse cardiac structural and functional outcomes in adults. For instance, hypertrophic remodeling of the cardiac tissues with increased collagen deposition and cross-linking was observed in adult male offspring exposed to prenatal hypoxia [10,11]. Male and female adult prenatal hypoxia offspring were also reported to have diastolic dysfunction and a substantially reduced cardiac recovery to ischemia/reperfusion insult [10,12,13,14]. However, the mechanisms by which intrauterine exposure to hypoxia can have long-term effects on the offspring’s cardiac function are not fully understood. 

One of the mechanisms could be mitochondrial dysfunction. The adult heart is a highly metabolic organ, where mitochondria occupy about 30% of the volume of the cardiomyocytes [15,16]. A large supply of cardiac energy is required to continuously pump blood through the body. Under normal conditions, about 95% of the adenosine triphosphate (ATP) that maintains cardiac function is provided by mitochondrial oxidative phosphorylation (OXPHOS) [17,18]. Electron flux through the system of protein complexes I to IV located in the inner mitochondrial membrane produces energy that drives proton outflow into the intermembrane space. This creates a proton gradient, which in turn allows protons to flow back into the mitochondrial matrix through ATP synthase (also known as complex V). The entry of protons into ATP synthase changes its conformation, allowing the phosphorylation of adenosine diphosphate (ADP) into ATP [19,20]. Reduced nicotinamide adenine dinucleotide (NADH) is oxidized to feed electrons by the NADH (N) pathway (complex I-linked), while the oxidation of succinate feeds electrons by the Succinate (S) pathway (complex II-linked). The N-pathway is initiated by NADH-linked substrates such as pyruvate, malate, and glutamate and runs through complex I, complex III, and complex IV. The S-pathway, on the other hand, is initiated by succinate and runs through complex II, while complex III and IV are common for both pathways. Complex V actively produces ATP when either the N- or S-pathways, or both, are active [21]. Inefficiency in these mitochondrial respiration pathways reduces the supply of ATP and leads to energy depletion.

The importance of mitochondria in sustaining cardiac function, along with their abundance in cardiac muscle [22], is such that mitochondrial dysfunction is often associated with cardiac pathologies. Indeed, changes in cardiac mitochondrial respiration were shown to be associated with hypertrophic cardiomyopathy [23], reperfusion injury following myocardial ischemia [24], diastolic dysfunction leading to heart failure [25,26], early development of heart failure [27], and hypertension in females during pregnancy [28]. For instance, a decreased OXPHOS capacity of the N-pathway was found in the hearts of patients with chronic heart failure (47–61 years old) [27], while the cardiac respiratory capacity of the S-pathway was reduced in a canine model of heart failure [26]. Moreover, hypertrophic cardiomyopathy was associated with reduced activity of the cardiac mitochondrial complexes I and IV in 6–18-month-old pigs [23], suggesting an inefficiency of the initiation of the N-pathway at complex I. A reduction in maximum respiratory capacity (indicating a decrease in the capacity of both N- and S-pathways) was found with the development of heart failure with preserved ejection fraction in the hearts of female mice 8–9 weeks old [25]. Moreover, hypertensive disorders of pregnancy were associated with a decrease in the capacity of the N-pathway in the hearts of pregnant rats [28]. These findings underline the importance of the proper functioning of the mitochondrial N- and S-pathways in the heart. A reduction in mitochondrial respiratory capacity will decrease the production of ATP and may alter cardiac metabolism. For instance, a reduced supply of ATP will interfere with the production of phosphocreatine necessary for maintaining the ATP reserve for myocardial contraction, leading to cardiac dysfunction [29,30,31]. Since offspring exposed to hypoxic conditions in utero were shown to be highly susceptible to cardiovascular diseases in adulthood [10,12,14,32,33,34,35], cardiac dysfunction in these offspring may be due to adverse changes in cardiac mitochondrial function. However, while associated with offspring cardiac dysfunction, very little is known about the impact of prenatal hypoxia on cardiac mitochondrial function in young adult offspring. A few recent studies reported impaired enzyme activities and protein expression of mitochondrial OXPHOS complexes in adult offspring exposed to prenatal hypoxia [36,37], suggesting cardiac mitochondrial function may be altered or impaired. However, a full understanding of how the dynamic changes in metabolic flux are affected as the electrons flow through the electron transport system is lacking.

The link between prenatal hypoxia and offspring cardiac dysfunction is believed to be the placenta. Fetal hypoxia is associated with placental oxidative stress and dysfunction, leading to a reduced supply of oxygen and nutrients to the fetus as well as the release of harmful factors into both the maternal and fetal circulation [38,39,40]. It was previously shown that in vitro-conditioned media containing placental stress-derived factors caused impairment in fetal neuron [38,41] and cardiomyocyte development [42]. Oxidative stress is a broad term that includes reactive oxygen species (ROS) produced within the mitochondria under pathological conditions in most mammalian cells. Placental mitochondrial content increases during pregnancy [43] and in complicated pregnancies, higher mitochondrial content of the placenta [44] increases ROS production [45,46,47,48]. Therefore, we have previously developed a therapeutic approach to treating the placenta using mitochondrial antioxidant MitoQ during pregnancy by encapsulating MitoQ into nanoparticles (nMitoQ). Encapsulation of MitoQ in the nanoparticles prevents potential off-target effects on the fetus as it results in the accumulation of nMitoQ in the placental labyrinth zone without crossing the placental barrier to the fetus [49]. In pregnancies complicated by prenatal hypoxia, this treatment with nMitoQ was previously reported to have both immediate and long-term beneficial and sex-specific effects on the offspring. For instance, while nMitoQ treatment in hypoxic dams increased placental efficiency and reduced placental oxidative stress in both sexes, only female placentae had an increase in oxygenation and a reduction in nitrosative stress after nMitoQ treatment compared to saline treatment. Moreover, placental angiogenesis was decreased by prenatal hypoxia in both sexes, while it improved with nMitoQ in the female placentae only [50]. nMitoQ also prevented hypoxia-induced changes in neurodevelopment [49] and cardiomyocyte maturation in male and female fetuses [42]. Moreover, long-term benefits of maternal nMitoQ treatment in prenatal hypoxia include improved cardiac morphology and diastolic function in the adult female offspring [14] and cardiac recovery post-ischemia in both male and female adult offspring [51]. However, the mechanisms by which nMitoQ treatment improves long-term cardiac function in young adult offspring exposed to prenatal hypoxia require further study.

The main objective of the current study was to assess whether in utero exposure to hypoxia impairs cardiac mitochondrial function in young adult offspring and if these changes can be prevented by our therapeutic intervention with nMitoQ. We hypothesized that cardiac mitochondrial respiration is impaired in young adult male and female offspring exposed to prenatal hypoxia and that this is ameliorated by nMitoQ treatment during pregnancy. Since placental stress was shown to have a different effect on male and female placentae, which was improved by nMitoQ treatment in a sex-specific manner [50], we hypothesized a sexually dimorphic effect of prenatal hypoxia and nMitoQ treatment on young adult offspring cardiac mitochondrial function. 

## 2. Results

### 2.1. Prenatal Hypoxia Decreased Cardiac OXPHOS Capacity in Female Offspring, Which Tended to Be Improved by Maternal nMitoQ Treatment

**Figure 1:**
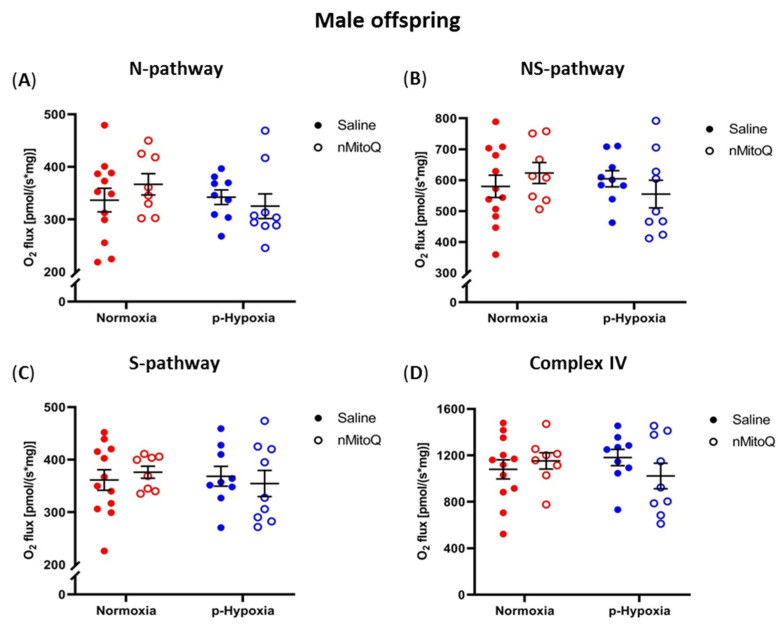
Effects of prenatal hypoxia and nMitoQ treatment on cardiac mitochondrial respiration in young adult male offspring. Oxidative phosphorylation (OXPHOS) capacity for the (**A**) Reduced nicotinamide adenine dinucleotide (NADH) pathway (N-pathway; substrates pyruvate, malate, and glutamate generating NADH, which feed electrons into complex I), (**B**) NADH- and succinate- (NS) pathways (maximum OXPHOS capacity when both NADH- and succinate- pathways are active), (**C**) succinate (S-) pathway (succinate feeding electrons into complex II after rotenone-mediated complex I inhibition), and (**D**) complex IV activity (ascorbate and TMPD feeding electrons into complex IV) were measured in the hearts of young adult (4-month-old) male offspring exposed to prenatal hypoxia (blue) or normoxia (red) after treatment with saline (closed symbols) or nMitoQ (open symbols). O_2_ flux is measured as [pmol/(s*mg)] where * stands for multiplication. Data were presented as mean ± SEM (*n* = 8–12 dams/group, 1–2 offspring/dam/group) and analyzed by two-way ANOVA.

**Figure 2:**
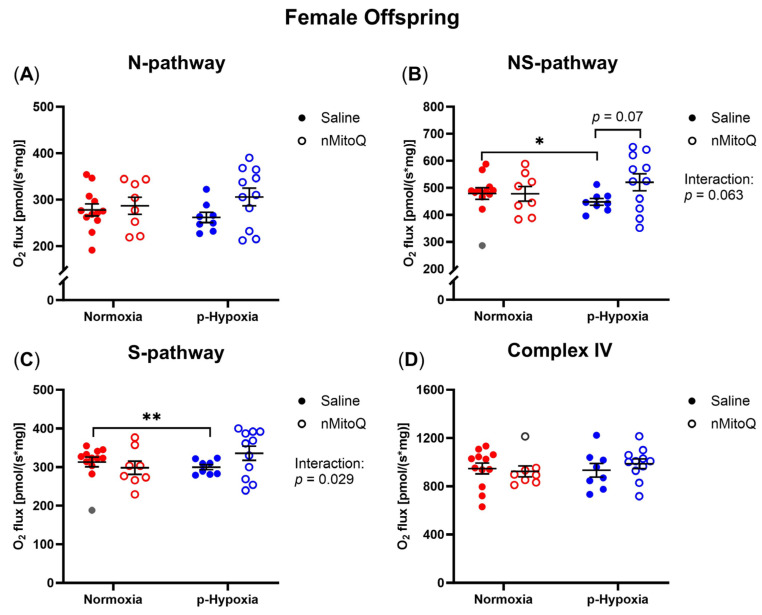
Effects of prenatal hypoxia and nMitoQ treatment on cardiac mitochondrial respiration in young adult female offspring. Oxidative phosphorylation (OXPHOS) capacity for the (**A**) NADH (N-) pathway (substrates pyruvate, malate, and glutamate generating NADH, which feed electrons into complex I), (**B**) NADH- and succinate- (NS) pathways (maximum OXPHOS capacity when both NADH- and succinate- pathways are active), (**C**) succinate (S-) pathway (succinate feeding electrons into complex II after rotenone-mediated complex I inhibition), and (**D**) complex IV activity (ascorbate and TMPD feeding electrons into complex IV) were measured in the hearts of young adult (4-month-old) female offspring exposed to prenatal hypoxia (blue) or normoxia (red) after treatment with saline (closed symbols) or nMitoQ (open symbols). Outlier data points are shown in gray. O_2_ flux is measured as [pmol/(s*mg)] where * stands for multiplication. Data were presented as mean ± SEM (*n* = 8–12 dams/group, 1–2 offspring/dam/group) and analyzed by two-way ANOVA with an unpaired two-tailed *t* post hoc test and Bonferroni correction (* *p* < 0.05, ** *p* < 0.01).

Mitochondrial respiration is presented as oxygen flux per tissue mass for the assessed pathways in hearts from male (Figure 1) and female (Figure 2) offspring. In the male offspring, compared to the normoxia group, prenatal hypoxia did not affect the cardiac OXPHOS capacity of the N-, NS-, and S-pathways, or complex IV (Figure 1A–D). Furthermore, maternal nMitoQ treatment did not alter the capacity of any of the OXPHOS pathways in male offspring (Figure 1).

In young adult female offspring, no effects of prenatal hypoxia or nMitoQ treatment were evident in the N-pathway (Figure 2A). However, prenatal exposure to hypoxia decreased the cardiac NS-pathway compared to the normoxic female offspring (Figure 2B, *p* = 0.025), which tended to be improved with maternal nMitoQ treatment (Figure 2B, *p* = 0.07). Prenatal hypoxia also decreased the S-pathway in the hearts of young adult female offspring compared to normoxic control females (Figure 2C, *p* = 0.009), while the reduction in the S-pathway in prenatal hypoxia-exposed females was no longer apparent with nMitoQ treatment (Figure 2C). No changes in cardiac complex IV activity were evident by prenatal hypoxia or nMitoQ treatment in young adult female offspring (Figure 2D). 

### 2.2. nMitoQ Treatment Increased OXPHOS Coupling Efficiency in Female Offspring Exposed to Prenatal Hypoxia

**Figure 3:**
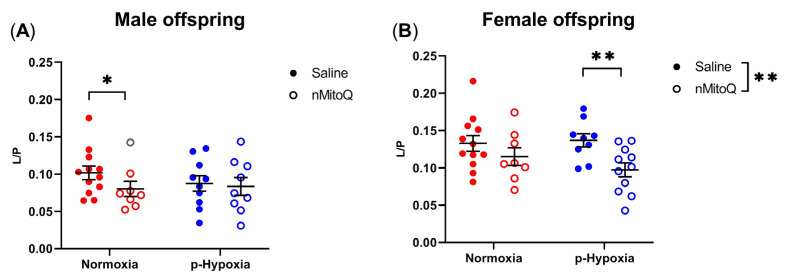
Effects of prenatal hypoxia and nMitoQ treatment on cardiac mitochondrial respiratory coupling efficiency in young adult male and female offspring. The OXPHOS coupling efficiency is shown as the ratio between LEAK respiration (L) and respiration with the NADH-linked substrates (P) pyruvate, malate, and glutamate without supplementation with ADP in the hearts of young adult (4-month-old) (**A**) male and (**B**) female offspring exposed to prenatal hypoxia (blue) or normoxia (red) after treatment with saline (closed symbols) or nMitoQ (open symbols). Outlier data points are shown in gray. Data were presented as mean ± SEM (*n* = 8–12 dams/group, 1–2 offspring/dam/group) and analyzed by two-way ANOVA with an unpaired two-tailed *t* post hoc test and Bonferroni correction (* *p* < 0.05, ** *p* < 0.01).

Non-phosphorylating LEAK respiration in the absence of ADP was assessed (L) and expressed over OXPHOS capacity with substrates feeding complex I (P), providing an expression of respiratory coupling efficiency independent of the mitochondrial content [21]. Prenatal hypoxia had no effect on OXPHOS coupling efficiency, shown as L/P ratio, either in male or female offspring cardiac tissue compared to normoxia control offspring (Figure 3). However, L/P was decreased (i.e., an increase in coupling) by maternal nMitoQ treatment in young adult normoxic male offspring (Figure 3A, *p* = 0.030) and prenatal hypoxia-exposed female offspring (Figure 3B, *p* = 0.008). 

### 2.3. No Differences in Mitochondrial Content between the Groups

**Figure 4:**
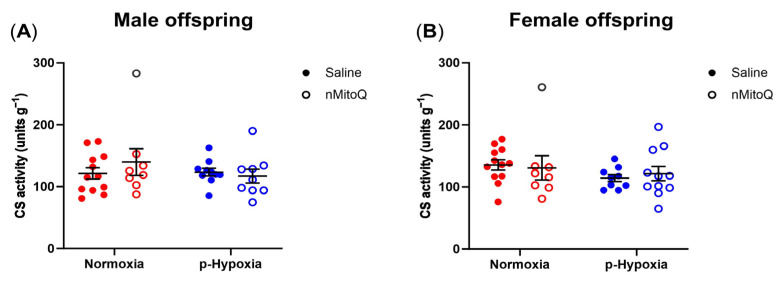
Effects of prenatal hypoxia and nMitoQ treatment on cardiac mitochondrial content in young adult male and female offspring. Citrate synthase activity was measured as a marker of mitochondrial content (expressed per g of tissue) in the hearts of young adult (4-month-old) (**A**) male and (**B**) female offspring exposed to prenatal hypoxia (blue) or normoxia (red) after treatment with saline (closed symbols) or nMitoQ (open symbols). Outlier data points are shown in gray. Data were presented as mean ± SEM (*n* = 8–12 dams/group, 1–2 offspring/dam/group) and analyzed by two-way ANOVA.

Based on previous studies, citrate synthase (CS) activity was used as a biomarker for mitochondrial content [21,52,53]. Neither prenatal hypoxia nor maternal nMitoQ treatment altered the cardiac mitochondrial content in young adult male or female offspring (Figure 4).

## 3. Discussion

In this study, we investigated the long-term effects of a common pregnancy complication, i.e., prenatal hypoxia, on cardiac mitochondrial function in young adult offspring and assessed the effects of an early (prenatal) intervention to prevent the impact of prenatal hypoxia on cardiac mitochondrial function later in life. We showed that prenatal hypoxia decreased the NS- and S-pathway OXPHOS capacities in the hearts of the female offspring only, which for the NS-pathway tended to be improved by nMitoQ treatment in utero. Contrarily, in the male offspring, there were no significant effects of prenatal hypoxia exposure or nMitoQ treatment on any of the OXPHOS pathway capacities. Cardiac mitochondrial L/P ratio, a measure of mitochondrial coupling, was not altered by prenatal hypoxia in either males or females; however, L/P was lowered by nMitoQ treatment in normoxic males and prenatally hypoxic females. Taken together, our findings suggest that in female offspring, in utero exposure to prenatal hypoxia induced a long-term impairment of cardiac mitochondria in the NS- and S-pathways, while maternal nMitoQ treatment tended to improve the maximum OXPHOS capacity and increase mitochondrial coupling.

We found sex-specific differences in the cardiac mitochondrial function of young adult offspring after exposure to prenatal hypoxia. In the female offspring only, a reduction in the maximum OXPHOS capacity, that is, when both the N-pathway (complex I-linked) and the S-pathway (complex II-linked) were active, was observed. The decrease in the maximum OXPHOS capacity was evident only after the addition of succinate and persisted as a reduction in the capacity of the S-pathway in prenatal hypoxia-exposed females compared to normoxia control females when complex I was inhibited. The lack of differences in CS activity and N-pathway capacity between the groups suggests that the observed pathway capacity changes were not due to changes in cardiac mitochondrial content or complex I activity (or enzymes and transporters upstream of complex I), but were likely due to components within the S-pathway, or, to be specific, due to changes in the complex II flux. Interestingly, under hypoxic conditions, it could be advantageous to decrease the use of the S-pathway to produce energy. This is because the phosphate/oxygen (P/O) ratio of the mitochondrial OXPHOS was shown to be 1.5 when succinate is oxidized to feed electrons through the S-pathway, whereas it is 2.5 when the NADH-linked pathway is used. In other words, for every oxygen atom reduced into water, 2.5 phosphates are incorporated into ATP in the N-pathway, whereas it is only 1.5 for the S-pathway [54]. Of note, a decrease in S-pathway activity may help to reduce ROS production through reverse electron transport to complex I during cardiac ischemia–reperfusion (I/R) injury [55,56]. However, without a secondary insult, a decrease in the S-pathway may decrease the overall energy production in the heart, thereby interfering with proper cardiac function, which may lead to left ventricular diastolic dysfunction [11]. In the hearts of adult female offspring in a mouse model of prenatal hypoxia, there was an increase in the oxygen flux through the complex I-linked pathway and through complex IV and consequently an increase in the maximum OXPHOS capacity that may have allowed for the optimization of energy efficiency in hypoxic conditions by limiting oxygen consumption [37]. In our study, however, there was only a decrease in S-pathway capacity with no observed compensation through improvement of the N-pathway capacity. The differences may be due to the animal model used and the severity and timing of the hypoxia exposure. For instance, Hellgren et al. (2021) used 14% O_2_ from GD 6–18 (equivalent to the 2nd and 3rd trimesters combined) [37], while we used 11% O_2_ from GD 15–21 (equivalent to the 3rd trimester of pregnancy). Therefore, the decrease in the S-pathway capacity we observed in the females without improvement of the N-pathway capacity suggests that cardiac mitochondrial respiration is impaired by prenatal hypoxia in a way that decreases the capacity and efficiency of energy production. This could have severe consequences for a highly metabolic organ such as the heart, as a decrease in the S-pathway may decrease ATP production and has been shown to be associated with the progression of heart failure [25,26]. Additionally, while a reduction in the S-pathway can be beneficial during cardiac I/R injury, it also means there will be a decrease in energy production during the I/R insult. This could impair cardiac recovery post-ischemia and could be one of the potential mechanisms behind the high susceptibility of prenatally hypoxic female offspring to cardiac I/R injury, as evidenced in our previous studies [12,51].

Interestingly, the decrease in maximum OXPHOS capacity in the hearts of the young adult female offspring exposed to prenatal hypoxia tended to improve with maternal nMitoQ treatment. In our previous studies, we showed that maternal nMitoQ treatment in prenatal hypoxia improved cardiac diastolic function [14] and cardiac recovery following an I/R insult [51] in adult female offspring. This attenuation in cardiac function in female offspring may thus be attributed to improved cardiac mitochondrial function. Furthermore, previous studies from our lab have also shown that nMitoQ treatment in prenatal hypoxia increased placental efficiency, decreased placental oxidative/nitrosative stress, and improved placental oxygenation in female placentae [50]. Our findings suggest a protective role for nMitoQ in female fetuses, likely through the placenta, which may attenuate mitochondrial function and normalize energy production in the hearts of young adult female offspring. Therefore, provided a dysfunctional placenta is known to release harmful factors that can impair fetal growth [39,42], cardiac development and function in the female offspring may have improved due to an amelioration of their placental conditions. 

The observed changes after hypoxia and/or nMitoQ treatment were specifically located in the S-pathway and may thus arise from alterations in the transport of succinate, the activity of complex II, or the complexes downstream of complex II (i.e., complexes III or IV). However, our data showed that complex IV activity remained unaltered between the groups in either male or female offspring cardiac tissue. Therefore, the decrease in S-pathway in prenatal hypoxia-exposed females may have been due to a decrease in either complex II or complex III activity. Since complex III is shared between the S- and N-pathways, a decrease in its activity would also have affected the capacity of the N-pathway, which was not observed. Thus, it appears that complex II could be responsible for the decreased S-pathway capacity after prenatal exposure to hypoxia. In fact, a previous report in a mouse model of prenatal hypoxia (14% O_2_ from GD 6–18) showed a decrease in mitochondrial complex II activity in the hearts of adult female offspring [37]. The exact mechanisms behind the changes in cardiac mitochondrial respiration in young adult female offspring are not known. A differential response of the male and female placentae to prenatal hypoxia has been shown with increased nitrosative stress in only female placentae [50], which may result in the dimorphic effects of prenatal hypoxia on male and female fetuses. Moreover, a reduction in the enzyme activity of complexes II and IV and mitochondrial oxidative capacity was found in embryonic cardiomyocytes due to defects in cardiac development [57]. Therefore, alterations in cardiac developmental programming due to prenatal hypoxia may result in a decrease in the complex II-linked S-pathway in female fetuses and may persist as long-term changes in cardiac mitochondrial function in young adult female offspring.

Our current study showed that prenatal hypoxia impacted mitochondrial function in the hearts of young adult female offspring only, without affecting the males. This was not expected, as previous studies reported a decrease in oxygen flux for the complex I-linked pathway [37] and a decrease in the activity of complex I [36] and complex IV [36,58] in the hearts of male offspring exposed to prenatal hypoxia. However, this difference in findings may have been due to the use of a mouse or guinea pig model and an extended duration of hypoxia (GD 6–18) [36,58]. A brief period (2–3 days) of normoxic exposure before birth may also have contributed to the differences in results [37]. Our findings were also unexpected because we and others have previously reported that exposure to intrauterine hypoxia led to adverse remodeling of the left ventricles, diastolic dysfunction [10], and reduced cardiac performance recovery following an I/R injury [12,51] in adult male offspring. Increased susceptibility to I/R injury was also found in adult female offspring exposed to hypoxic conditions in utero [12]. Thus, our current data suggest that although adult male and female offspring exposed to prenatal hypoxia were previously found to have similar impairments in their cardiac function [11,12], the mechanisms are different. While cardiac dysfunction in young adult female offspring may be (in part) attributed to a reduced capacity of the cardiac mitochondrial NS- and S-pathways, these were not impacted by prenatal hypoxia in the male offspring. However, it may be speculated that prenatal hypoxia alters the activity or expression of mitochondrial complex proteins in males or may even lead to changes in mitochondrial dynamics or ROS production without effects on the mitochondrial OXPHOS that were assessed in the current study. This highlights sex-specific mechanisms leading to later-life cardiac function/dysfunction after prenatal hypoxia. 

Measurement of the LEAK respiration allows for an estimation of the mitochondrial respiration uncoupled from ATP production when protons cross the inner mitochondrial membrane, bypassing ATP synthase. It compensates for the proton and electron leak, proton slip, and cation cycling across the inner mitochondrial membrane at resting state [21]. A range of 0.0 to 1.0 is used to identify mitochondrial coupling from our L/P values, with 0.0 denoting a fully coupled system and 1.0 denoting a fully uncoupled system. Our data showed that all the L/P values are below 0.22, indicating high respiratory coupling efficiency in the hearts of all offspring groups, and this was not affected by prenatal hypoxia. Therefore, it may be speculated that these young adult offspring hearts are efficiently consuming oxygen to generate energy in the form of ATP, which was not altered by intrauterine exposure to a hypoxic environment. Interestingly, maternal nMitoQ treatment further decreased the L/P ratio, thereby increasing mitochondrial coupling, in normoxic male and prenatal hypoxia-exposed female offspring. A decrease in the L/P ratio will increase the efficiency of ATP production and was found to be coupled with an increasing trend in maximum OXPHOS capacity in female prenatal hypoxia-exposed offspring with nMitoQ treatment. This may help to meet the increased energy demand of the heart when required and can contribute to improved cardiac functional outcomes. For instance, an increased workload subsequently increases cardiac energy consumption in pressure-overload cardiac hypertrophy [59]. Therefore, prenatally hypoxic female offspring would be able to meet the excess cardiac energy demand during pressure overload if treated in utero with a placenta-targeted antioxidant. Further studies are necessary to determine whether the decrease in the L/P ratio found in the current study is indeed associated with a significant increase in energy production in the hearts of the prenatally hypoxic female offspring. However, the decrease in L/P in normoxic males has little importance since nMitoQ treatment is not necessary for normal pregnancies. 

To conclude, prenatal/fetal hypoxia severely impacts the offspring’s later-life cardiovascular health, which could be prevented by prenatal intervention strategies. Here, we showed that intrauterine exposure to hypoxia induced long-term alterations in cardiac mitochondrial respiration in female offspring and that prenatal treatment strategies may be used as a possible intervention to improve cardiac mitochondrial respiration in young adult female offspring. Similar treatment strategies were also reported to be effective in improving cardiac function in the male offspring, but this appears to be through other cardiac mechanisms. Our data highlight the need for sex-specific treatment strategies for improving the health of offspring from complicated pregnancies.

## 4. Materials and Methods

### 4.1. Ethics Approval

This study was performed according to experimental protocols approved by the University of Alberta Health Sciences Animal Policy and Welfare Committee, under the guidelines of the Canadian Council on Animal Care and the Guide for the Care and Use of Laboratory Animals (AUP #3693). 

### 4.2. Prenatal Hypoxia Rat Model and Experimental Design

Three-month-old male (for breeding) and female Sprague Dawley rats were purchased from Charles River (Kingston, NY, USA) and were housed under standard housing conditions in a 10:14 h light/dark cycle with a temperature of 22 °C and 60% humidity in the Animal Care Facility at the University of Alberta. A standard chow diet and water were provided ad libitum. After one week of acclimatization, female rats were housed with males overnight. Gestational day (GD) 0 of pregnancy was defined by the presence of sperm in the vaginal smear the next morning. As reported in previous studies [14,45,50,51], pregnant dams were injected with a single dose (100 µL) of either saline (control) or nMitoQ (125 µmol/L) on GD 15 by the tail vein and randomly assigned to a hypoxia or normoxia group (*n* = 8–12 dams/group). For the production of nMitoQ, MitoQ was adsorbed with an efficiency of about 30% on nanoparticles made by conjugating poly-γ-glutamic acid and L-phenylalanine ethylester, as previously described [49]. The normoxia group dams were single-housed at atmospheric oxygen (21% O_2_) throughout pregnancy. The dams in the hypoxia group were placed in a hypoxic chamber with 11% O_2_ from GD 15–21. Dams were removed from the chamber on GD 21 (GD 22 = term) and allowed to give birth in standard housing conditions. After birth, litters were reduced to 4 males and 4 females to standardize postnatal conditions. Offspring were weaned at postnatal day 21, and same-sex littermates were double housed and aged to 4 months in standard housing conditions. After 4 months, male and female offspring (1 or 2 offspring/sex/dam) were anesthetized using isoflurane and euthanized by excision of the heart. The left ventricles of the heart were collected and used immediately for high-resolution respirometry.

### 4.3. High-Resolution Respirometry

Respirometry measurements were performed using an Oxygraph-2k system (OROBOROS Instruments, Innsbruck, Austria). The preparation of cardiac tissues for respirometry included permeabilization of the myocardial fibers, as described previously [27]. In short, left ventricular tissues were rinsed in an ice-cold biopsy preservation solution (BIOPS; Ca-EGTA 10 mmol/L, free calcium 0.0001 mmol/L, imidazole 20 mmol/L, taurine 20 mmol/L, K-MES 50 mmol/L, DTT 0.5 mmol/L, MgCl_2_ 6.56 mmol/L, ATP 5.77 mmol/L, phosphocreatine 15 mmol/L, pH 7.1 at 0 °C). Next, a two-step approach to tissue permeabilization was performed: first manually by slowly separating the cardiac fibers using a pair of forceps, then with saponin (0.05 mg/mL) in ice-cold BIOPS for 30 min with gentle agitation on a plate shaker. The fibers were then transferred to mitochondrial respiration medium 05 (MiR05; EGTA 0.5 mmol/L, MgCl_2_ 3 mmol/L, lactobionic acid 60 mmol/L, taurine 20 mmol/L, KH_2_PO_4_ 10 mmol/L, HEPES 20 mmol/L, sucrose 110 mmol/L, and fatty acid-free BSA 1 g/L, pH 7.1) and washed for 10 min with continuous agitation on ice. Following permeabilization, tissues collected from each rat were divided into triplicates and then added to separate high-resolution respirometry chambers containing 2 mL MiR05 at 37 °C for measuring mitochondrial respiration. The variation among the triplicates was 0.17 ± 0.01 mg (mean ± SEM). 

**Figure 5:**
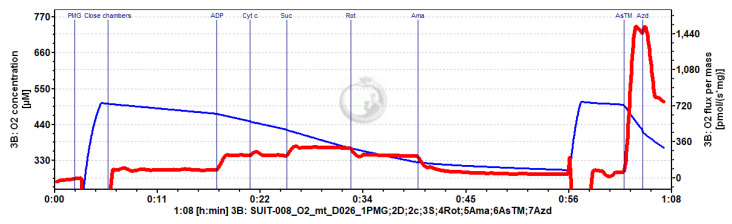
Original representative tracing of mitochondrial oxygen consumption in the heart of a young adult female exposed to prenatal hypoxia. See the methods section for a detailed protocol. Level of oxygen in the chamber (blue) and OXPHOS capacity measure as O_2_ flux per mass (red). Abbreviations: PMG, pyruvate, malate, and glutamate; Cyt c, cytochrome c; Suc, succinate; Rot, rotenone; Ama, Antimycin A; AsTM, ascorbate and TMPD; Azd, azide.

Mitochondrial respiration was measured in the LEAK and OXPHOS states using the carbohydrate substrate–uncoupler–inhibitor titration (SUIT) protocol (Figure 5). During the respirometry measurement, oxygen limitation in fibers was avoided by maintaining an oxygen level between 240 and 500 μmol/L O_2_. LEAK respiration was measured following the addition of pyruvate (5 mmol/L), malate (5 mmol/L), and glutamate (10 mmol/L) in the absence of ADP. Next, OXPHOS capacity through the N-pathway (complex I-linked) was stimulated by the addition of ADP (2.5 mmol/L). The addition of cytochrome c (0.01 mmol/L) then allowed for the assessment of the integrity of the outer mitochondrial membrane. A 0–21% increase in flux with cytochrome c, with only 10% of total experiments experiencing a higher than 15% increase in respiration, indicated that the integrity of the outer mitochondrial membrane was preserved during tissue preparation. Cytochrome c did not differ among the groups (data not shown. Succinate (10 mmol/L) was then added to assess the maximum OXPHOS capacity with convergent electron flow from complex I and complex II, the NADH + succinate (NS-) pathway. Subsequently, complex I was inhibited with rotenone (1 μmol/L) to measure the OXPHOS capacity of the S-pathway only (complex II-linked). After which, the flux through complex III (Ubiquinol-cytochrome c oxidoreductase) was inhibited with antimycin A (5 μmol/L) to correct for the non-mitochondrial residual oxygen consumption (ROX). Finally, complex IV (cytochrome c oxidase) activity was measured with ascorbate (2 mmol/L) and N,N,N′,N′ -tetramethyl-p-phenylenediamine (TMPD, 0.5 mmol/L). The chemical background obtained after inhibition of complex IV with sodium azide (100 mmol/L) was subtracted from the complex IV activity. After the titration protocol, cardiac fibers were collected from the oxygraph chambers and homogenized on ice for 30 s with a conical glass tissue grinder, aliquoted, and stored at −80 °C for the measurement of CS activity. 

Data on mitochondrial respiration were analyzed using the Datlab software (OROBOROS Instruments, Innsbruck, Austria). The OXPHOS capacity of the mitochondrial respiration pathways and complex IV was represented as oxygen flux per tissue mass. LEAK respiration was calculated as the Flux Control Ratio (FCR) by normalizing the values to the maximum OXPHOS capacity. All triplicates from each heart were averaged and represented as a single data point in all experimental groups.

### 4.4. Mitochondrial Content

The maximum activity of CS reflects mitochondrial content [52]. Frozen cardiac muscle fiber homogenates were thawed on ice, vortexed, and transferred to a 1 mL cuvette with 5,5′dithiobis-2-nitrobenzoic acid 0.1 mL (DTNB, 0.1 mmol/L) (ε: 13.6 mL cm^−1^ μmol^−1^), Triton X-100 0.25%, oxaloacetate 0.5 mmol/L, acetyl-CoA 0.31 mmol/L, and Tris-HCl 100 mmol/L. The absorbance was measured for 300 s at 412 nm following the reduction in DTNB using a UV/Vis spectrophotometer (Ultrospec 2100 pro; Biochrom Ltd., Cambridge, UK). The CS data were normalized to the mass of the tissue in the homogenate.

### 4.5. Statistical Analyses

Statistical comparisons were conducted using GraphPad Prism 9 (GraphPad Software, San Diego, CA, USA). Grubbs’ test was used to remove significant outliers from the final analyses. Data were then analyzed by two-way ANOVA to assess the main effects of prenatal hypoxia and nMitoQ treatment, with an unpaired two-tailed *t* post hoc test and Bonferroni correction. Data were presented as mean ± SEM; *p* < 0.05 was considered statistically significant.

## Data Availability

Data supporting the reported results are contained within the article.
